# Mental practice-based rehabilitation training to improve arm function and daily activity performance in stroke patients: a randomized clinical trial

**DOI:** 10.1186/1471-2377-8-7

**Published:** 2008-04-11

**Authors:** Jeanine A Verbunt, Henk AM Seelen, Feljandro P Ramos, Bernard HM Michielsen, Wim L Wetzelaer, Martine Moennekens

**Affiliations:** 1 Rehabilitation Foundation Limburg, Hoensbroek, The Netherlands; 2 Department of General Practice, Maastricht University, Maastricht, The Netherlands; 3 Department of Rehabilitation Medicine, Academic Hospital Maastricht, Maastricht, The Netherlands; 4 Care And Public Health Research Institute, Maastricht University, Maastricht, The Netherlands; 5 Department of Rehabilitation Medicine, Atrium Medical Center, Heerlen, The Netherlands; 6 Department of Rehabilitation Medicine, VieCuri Medical Center, Venlo, The Netherlands

## Abstract

**Background:**

Over 50% of patients with upper limb paresis resulting from stroke face long-term impaired arm function and ensuing disability in daily life. Unfortunately, the number of effective treatments aimed at improving arm function due to stroke is still low. This study aims to evaluate a new therapy for improving arm function in sub-acute stroke patients based on mental practice theories and functional task-oriented training, and to study the predictors for a positive treatment result. It is hypothesized that a six-week, mental practice-based training program (additional to regular therapy) targeting the specific upper extremity skills important to the individual patient will significantly improve both arm function and daily activity performance, as well as being cost effective.

**Methods/design:**

One hundred and sixty sub-acute stroke patients with upper limb paresis (MRC grade 1–3) will participate in a single-blinded, multi-centre RCT. The experimental group will undertake a six-week, individually tailored therapy regime focused on improving arm function using mental practice. The control group will perform bimanual upper extremity exercises in addition to regular therapy. Total contact time and training intensity will be similar for both groups. Measurements will be taken at therapy onset, after its cessation and during the follow-up period (after 6 and 12 months). Primary outcome measures will assess upper extremity functioning on the ICF level of daily life activity (Wolf Motor Function Test, Frenchay Arm Test, accelerometry), while secondary outcome measures cover the ICF impairment level (Brunnstrom-Fu-Meyer test). Level of societal participation (IPA) and quality of life (EuroQol; SS-Qol) will also be tested. Costs will be based on a cost questionnaire, and statistical analyses on MAN(C)OVA and GEE (generalized estimated equations).

**Discussion:**

The results of this study will provide evidence on the effectiveness of this mental practice-based rehabilitation training, as well as the cost-effectiveness.

**Trial registration:**

Current Controlled Trials [ISRCTN33487341)

## Background

The incidence of stroke in the Netherlands was 2.2/1000 in 2000 [1]. Over 50% of patients with upper limb paresis resulting from stroke face long-term impaired arm function and a ensuing disability in daily life [2]. Although early rehabilitation treatment in the first phase of stroke is currently advised in clinical guidelines, little evidence is available on such treatment for improving arm/hand function in the same period [3]. Recently, constrained induced movement therapy (CIMT), in which the affected arm must be used due to immobilizing the other, appears to have had a positive effect on arm/hand function in sub-acute stroke patients [4]. However, even long after the stroke only small arm function improvements are reported in a select minority of patients, which stands in contrast to the far better recovery of leg function.

Some studies have shown task-oriented treatment to positively affect arm function recovery in stroke patients [5]. More recently, 'movement imagery' has emerged, targeting the cognitive processes associated with enhanced motor performance and specific skilled movements in healthy persons. Regularly applying this technique in training and competition is called 'mental practice'. Sports psychology research has shown that mental practice can optimize athletes' execution of movement and acquisition of new, skilled behaviors [6,7]. Moreover, combining mental practice training principles with active movement training appears more beneficial than mental practice training alone [6].

In neuroscience, interest in movement imagery has grown in line with improvements in brain mapping techniques. Results from brain imaging experiments suggest that executed and imagined movements share a common neural substrate [8-10]. This has led to the hypothesis that mental practice may contribute to the activation of neural loops and movement patterns for which the brain has a kind of motor print [11]. Mental practice might be used alongside physical rehabilitation in patients with neurological disorders and will probably be most effective in the early recovery stage during which the reorganization of brain patterns is most prominent [10].

The possible benefits of movement imagery on motor performance in acute and chronic stroke patients have been investigated in several studies. Page et al., in a small feasibility study using movement imagery in stroke patients, found a marked improvement in their arm function compared to that of the control group [12]. Similar effects in the training of neglect and motor skills have been reported by Smania et al. and Yoo et al. [13,14]. It seems, then, that although the brain is damaged by stroke, the ability to train using mental practice is retained.

The effects of mental practice on small chronic stroke groups have been reported in several studies [14-22] using different mental practice techniques and intensities. In 2006, Braun et al. presented a systematic review focusing on effectiveness of mental practice training in improving upper extremity functioning, but drew no definite conclusions except that further research using clear definitions of mental practice content and standardized outcome measurements are needed [21]. In 2007, based on a randomized controlled trial for chronic stroke patients, Page et al. confirmed the short-term effectiveness of mental practice in improving upper extremity function [23].

Only one article has reported on the long-term effect of mental practice in stroke patients. This was based on an evaluation conducted just one month after training; the conclusions were positive [22]. The effectiveness of mental practice in stroke, however, is likely influenced by factors such as the ability to perform motor imagery, cognitive functioning, gender, handedness, dysphasia, precise lesion location and time elapsed since the stroke [10,24]. Whether these factors are indeed predictive of therapy outcome and whether mental practice is also effective over the long term in sub-acute stroke patients is currently unknown.

Based on the latest body of evidence, it is hypothesized that mental practice applied in a training regime involving arm function tasks will lead to a significant and long-lasting improvement in stroke patients' arm function. Furthermore, since rehabilitation depends upon the learning of new behaviors (this is associated with brain plasticity), the treatment should include much repetition [2], start early after the stroke [2], be personally rewarding to the patients and provide as much experience as possible of various activities.

### Aims

The aim of the proposed research project is to systematically investigate the therapeutic potential of a mental practice-based therapy in the (partial) restoration of arm/hand function in sub-acute stroke patients. This aim has led to the following research questions:

1. Does a six-week, mental practice-based rehabilitation regime for patients with upper extremity paresis in the sub-acute stroke phase improve arm function and daily activity performance as compared to usual care?

2. What are the prognostic factors for a good therapy outcome?

3. Is a regime such as that described in (1) cost effective as compared to usual care?

## Methods/Design

### Study design

**Figure 1:**
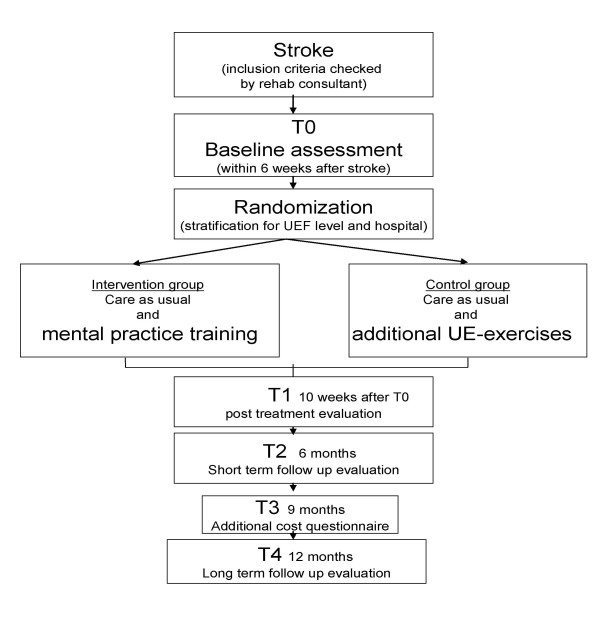
The design of the study.

A multi-centre, single-blinded, randomized controlled trial will be conducted to evaluate the effects of six weeks of mental practice-based treatment on arm function in unilateral stroke patients. The evaluation will span the full post-stroke year. Assessment will take place upon entry to the study; post-treatment and follow-up assessments will be performed on three moments during the year (see Figure 1 flow diagram). The Study protocol was approved by the Medical Ethical Committee of the Rehabilitation Foundation Limburg. The study will start in March 2008 and will last till September 2010.

### Setting

Patients from rehabilitation departments in the Hoensbroek rehabilitation centre as well as regional hospitals in South Limburg, the Netherlands, will be included. Patients can enter both the intervention and control training groups.

### Study population

Patients who meet the inclusion criteria in Table 1 will be referred by a consultant in rehabilitation medicine. The sample size calculation (two groups and two-sided testing) was performed using data derived from Bonifer et al.'s [25] study in which stroke patients had a mean Wolf Motor Function Score on the functional ability scale of 3.91 with a standard deviation of 1.11. An assumed clinically relevant difference between groups of at least 10%, an alpha of 0.05 and a power (1-β) of 0.85 would necessitate 145 patients. With an estimated follow-up loss of approximately 10%, 160 participants (2 * 80) are thought to be necessary.

**Table 1:** Inclusion criteria

- first stroke
- post-stroke time of 2–6 weeks
- clinically diagnosed central paresis of arm/hand with elbow flexor strength MRC grade 1–3
- age 18–85
- no severely impaired cognition
- no severe neurological, orthopedic, rheumatoid or cardiac impairments prior to stroke
- no severely comprehension-impaired communication

### Recruitment and randomization

Patients admitted to the participating institutions will be evaluated on the basis of the inclusion criteria by a consultant in rehabilitation medicine involved in treatment within three days of admission to a stroke service. Time lapsed since the stroke occurred should preferably be less than two weeks. If the patient is still unconscious or incapacitated at this time, inclusion may take place as appropriate within the ensuing four weeks. Standard informed consent procedures will be used. Participants will then be randomly allocated to either the intervention or control group, with the computerized (block) randomization scheme including pre-stratification according to two variables: MRC scale of the elbow flexor (dichotomized in score 1 or scores 2–3); and participating hospital. Two sets of opaque, numbered envelopes will be prepared for each site (one each for MRC score 1 and MRC scores 2–3) containing cards indicating the allocated group. When a new patient is registered, a card will be extracted and the relevant occupational therapist informed of the group allocation.

### Intervention and control group

In accordance with Dutch stroke rehabilitation guidelines, the intervention group patients will receive their regular therapy [3] and additional mental practice-based arm function training. This training will be supervised by the rehabilitation team occupational therapist. After baseline measurements are performed, patients will be familiarized with the mental practice-based therapy during the first session. The patient is educated as to basic imagery principles and the importance of regular imagery training in increasing therapy success. During the first week, the patient will be taught how to use the mental practice techniques to improve arm function by the occupational therapist. A training task tailored to the functional level of the individual patients will be selected by the occupational therapist.

Five different mental practice training tasks derived from the Frenchay Activities Index [26] are available, with gradually increasing difficulty. For all tasks, a training DVD will guide the patient. Each DVD is programmed in three steps starting with a relaxation task to focus attention. Performance of all activities is shown from a 1^st ^person perspective (an 'over-the-shoulder' view). In the first step, correct task performance is shown on screen combined with a verbal explanation. Five repetitions are given. In the second step, task performance is repeated on screen but without verbal explanation; patients are asked to mentally practice the movement. If they are able to actually perform (part of) the task, they may do so concurrently with the imagination of the movement. Again, five repetitions are given. In the third step, no guidance during task performance is given except a visual and verbal cue indicating the end of the task performance over five repetitions. Patients already familiar with this task can immediately start on step two.

DVDs are available for every task for right- and left-handers. Patients must practice at least three times a day for ten minutes each session. During the intervention period, functional arm/hand progress will be evaluated by the occupational therapist every two weeks. If functional level improves, a new task will be chosen and the DVD changed. The total intervention lasts six weeks.

Patients in the control group receive therapy as usual. In addition, they will be instructed to practice additional bimanual upper extremity techniques based on conservative neurodevelopmental (NDT) principles [27]. These exercises are a part of the usual training program, and, in clinical practice, have been accepted as conventional therapy. However, recent studies have questioned NDT's additional value in stroke patients [28,29]. The control group patients will receive a booklet explaining all tasks and be instructed to practice for ten minutes at least three times a day. Every two weeks, the home-based training sessions will be evaluated by the team's occupational therapist. Total contact time with the occupational therapist in the intervention and control groups will be equal.

### Data collection

Measurements will be taken upon entry to the study (T0 = baseline) and 6 weeks (T1 = post treatment); 6 months (T2 = short-term follow-up); 9 months (T3 additional cost questionnaire) and 12 months (T4 = long term follow up) after T0. They will be performed by a rater blinded for therapy modality, and administered wherever the patient is at the time. A blinding check will be performed after each of the four measurement sessions.

### Outcome measures

Outcome measures and assessment moments are presented in table 2.

**Table 2:** Outcome domains and assessment instruments and moments

**Domain**	**Assessment instrument**	**Abbr.**	**T0**	**T1**	**T2**	**T3**	**T4**
Medical information	- Brain lesion		X				
	- Type of stroke		X				
	- Co-morbidity		X				
Basic level of functioning	- Barthel score	BI	X		X		X
	- Frenchay Activity Index	FAI	X*	X	X		X
Cognitive functioning	- Cognitive Log	Cog-log	X				
	- Vividness of Movement Imagery Q.	VMIQ	X				
	- Credibility/Expectancy Q.	CEQ	X				
UEF level of impairment	- Brunnstrom-Fugl-Meyer test	FM	X	X	X		X
	- Strength: part of WMFT	WMFT _str_	X	X	X		X
	- Spasticity: Tardieu scale	MTS	X	X	X		X
UEF level of activity	- Wolf Motor Function Test	WMFT	X	X	X		X
	- Frenchay Arm Test	FAT	X	X	X		X
	- Accelerometry	ACC	X	X	X		X
UEF level of participation	- Impact on Participation and Autonomy questionnaire	IPA					X
	- Stroke-Specific Quality of Life	SS-Qol					X
	- EuroQol	EO-6D		X	X		X
Cost	- Cost questionnaire	CQ	X	X	X	X	X
Process evaluation	- Diary		X				

T0 = baseline

UEF = upper extremity functioning

T1 = 10 weeks after T0

T2 = 6 months after T0

T3 = 9 months after T0

T4 = 12 months after T0

* The FAI on T0 retrospectively assesses the pre-morbid activity level

### Demographic and medical variables

The following socio-demographic variables will be recorded: age, sex, living situation and educational level. The medical variables include systematically scored, stroke-related neurological variables: lesion site, stroke type (hemorrhagic or ischemic), paresis level and co-morbidity. In the questionnaire, information on demographical variables is retrieved. Medical variables as presented above are derived from the medical file of the referring consultant in rehabilitation medicine. Patients will be asked for permission to use this information of their medical file in the informed consent procedure.

### Cognitive functioning

The following aspects of cognitive functioning were measured.

#### General level of cognitive functioning

The Cognitive Log (Cog-log) is a 10-item cognitive screening instrument which measures higher neurocognitive processes including orientation, memory, concentration and executive skills [30].

#### Ability to imagine motor acts

To asses participants' ability to imagine motor acts, the Vividness of Movement Imagery Questionnaire (VMIQ) will be used [31]. Individuals are required to rate the vividness of their imagery on a 5-point scale (1 = as perfectly clear and vivid as normal vision, 5 = no image at all – only a vague awareness of thinking about the movement) for 24 different movements from both an external (i.e. watching somebody else) and internal (doing it yourself) perspective. VMIQ scores have been linked to improvements in motor skills [31].

#### Credibility and expectancy of treatment

The Credibility/Expectancy Questionnaire (CEQ), a simple scale for measuring rationale credibility and treatment expectancy in clinical outcome studies will be used. The credibility and expectancy of mental practice and control training is scored by the patient. This questionnaire demonstrated high internal consistency and good test-retest reliability [32].

### Upper extremity functioning

Physical functioning will be assessed on three different levels, in accordance with the International Classification of Functioning [33]. Patients' performance on impairment, activity and participation levels will be assessed.

1. *Upper extremity functioning at the impairment level *will be assessed using two instruments, described below.

*1.1 *The arm section of the Fugl-Meyer Test (FMT) contains three different domains: (a) motor function (24 items; scores range from 0 to 66), (b) sensation (6 items; scores from 0 to 12) and (c) passive joint motion/joint pain (12 items, scores from 0 to 48)[34]. Each item is scored on a 3-point ordinal scale. The Fugl-Meyer Assessment of the Upper Extremity (FMA-UE) is the most widely used clinical assessment of post-stroke upper extremity impairment [35] and showed a very high inter-rater and test-retest reliability (ICC > 0.95) [35,36].

*1.2 *To score spasticity level, the Tardieu Scale will be used. The affected limb is passively moved through range at two velocities – i.e., as fast and as slow as possible (VI). At both velocities, the quality of the muscle reaction to stretch is assessed for each muscle group, and the angle at which the muscle reaction occurred is measured with a hand-held goniometer after the cessation of movement. Spasticity will be scored from 0–4 during the fast stretching velocity. In patients with severe brain injury and impaired consciousness, the Modified Tardieu Scale provides higher test-retest and inter-rater reliability than the Modified Ashworth Scale and may therefore provide a more valid spasticity scale for adults [37].

*2. Upper extremity functioning at the activity level *will measured using the following three instruments.

*2.1 *The Wolf Motor Function Test (WMFT) test contains 15 timed and 2 strength tasks (lifting of weighted limb and grip strength), ranging from simple to complex and administered sequentially to each upper extremity while controlling for patient positioning. Trained observers rate quality of movement using a 6-point functional ability scale (0 = no attempt; 5 = normal movement). Strength and performance time will be recorded by the test administrators. The psychometric properties of the WMFT appear solid [4,38-40].

*2.2 *The Frenchay Arm Test (FAT) will be used to assess the degree to which the patient is able to actually perform arm hand functions and tasks. It includes an evaluation of performance on five different tasks, and has shown good reliability and validity in stroke patients [26].

*2.3 A*ccelerometery (ACC) will be used to assess upper extremity use in a daily life situation. Patients will wear a 'watch' with an accelerometer inside (Actiwatch [41]) on both wrists for three days, during waking hours. This Actiwatch includes two uniaxial piezoresistive accelerometers used to record the amount of movement based on accelerations. Within both watches the rectified and integrated acceleration from two directions over one minute are registrated. The number of occasions on which this signal exceeds a predefined threshold is then calculated. The outcome is expressed in counts per minute, based on wrist accelerations. Data collection will continue uninterrupted for three days, with output stored in a data memory chip within the accelerometer and read out by a computer after this time.

The Actiwatch has shown good validity for assessing daily life activities [41]. In stroke, several studies have shown that accelerometry provides an objective, real-world index of arm activity with strong psychometric properties [42-45]. In the present study, an activity index will be calculated as a ratio between the activity of the impaired arm movement and that of the unimpaired arm movement. Uswatte et al. found that in stroke patients, a ratio score controls adequately for variations in overall levels of physical activity, and is a reliable and valid real-world measure of upper extremity treatment outcome [39].

*3. Functional performance on the level of participation in society and perceived quality of life *will be measured using three methods.

*3.1 *The Impact on Participation and Autonomy (IPA) questionnaire. This 39-item questionnaire focuses on two aspects of participation: the perceived participation level in different domains, and the problems encountered [46,47].

3.2 The Stroke-Specific Quality of Life (SS-Qol) scale. This includes an overall evaluation of the patient's quality of life compared with their pre-stroke state. Twelve domains are included: personality, energy, language, mobility, vision, upper extremity function, thinking, mood, work/productivity, self care and family and social roles [48].

*3.3 *The EuroQol 6D (EQ-6D). This is a generic measure scoring quality of life using six domains: mobility, self-care, usual activities, pain/discomfort, anxiety/depression and cognition. Each domain consists of three answer possibilities. Participants are also to rate their current health state on a visual analogue scale from 0 to 100 [49].

In brief, primary outcome measures for the evaluation of upper extremity functioning focus on the activity level (WMFT, FAT, ACC), whereas secondary outcome measures focus on both the impairment (FMT) and participation level (IPA, SS-Qol).

### Process evaluation

After completing the intervention, participants will be asked to evaluate it by way of a questionnaire. In addition, treatment compliance in both groups will be monitored using diaries recording the daily amount of time patients spend on mental practice training (intervention group) and NDT-based upper extremity training (control group). Compliance can be an important issue in studying patients in the sub-acute stroke phase [10]: poor compliance can negate interpretation of the outcome as reflecting the therapy's effectiveness. Through diary use, however, compliance is rendered objective. The intervention process will also be evaluated based on information provided by occupational therapists, who will record the following items: the number of consultations, the provision of information per consultation, the kind of problems presented, completion of the intervention according to protocol and reasons for non-compliance (where applicable).

### Determination of costs

Both direct non-medical and indirect costs will be measured using a cost questionnaire administered to the participants or their proxies at the time of the T0-T4 assessments [50]. An additional cost questionnaire will be sent to the patient at 9 months. With society's perspective for the economic evaluation, all relevant rehabilitation costs will be determined using a micro-costing approach. This means that the unit size of the resource used will be multiplied by the respective unit cost across patients to estimate the aggregate cost for society [51]. Healthcare resources used in administering the intervention as well as direct medical costs would include contact with professionals (e.g., physicians, therapists and rehabilitation nurses or formal caregivers), equipment and materials (e.g., orthoses), assessment tools, and medications (particularly anti-spasmodics and muscle relaxants). Along with allocated overhead costs, these measures will determined through the institutions' administrative records.

Direct non-medical costs include patient times, transportation and out-of-pocket expenses not covered by insurance. Indirect costs would cover productivity losses and informal care by the family or significant others. In line with recommendations in the Dutch manual for costing in economic healthcare evaluations, standardized costs will be used [52]. Medications will be valued based on the Daily Defined Dosage (DDD) price list from the Dutch Pharmacotherapeutic Compass [53]. Productivity losses will be valued using the friction cost method, which bases calculations on the 'friction period,' or the time needed to replace a sick employee. Informal care (considered unpaid work) would be valued using shadow prices.

### Statistical analysis

#### Effect evaluation

The number of dropouts (patients who prematurely end their participation) and follow-up losses in both training groups will be reported based on descriptive data. Baseline characteristics of compliant and non-compliant participants will be compared. Before examining the intervention's effectiveness, possible differences between the two groups will be determined by comparing baseline characteristics using independent sample T-tests (normal distribution) or Mann Whitney U-tests (non-normal distribution) in the event of continuous variables. In cases of dichotomous variables, a chi-square test will be used.

The effects of mental practice-based therapy on arm function outcome will be evaluated using MAN(C)OVA and GEE (generalized estimated equations) analyses. Deviations from the therapy protocols will be analyzed according to the 'intention-to-treat' principle. If either T2 or T4 data are missing, the 'last-observation-carried-forward' principle will be used. Losses of T0 and/or T1 data will lead to exclusion, and an additional participant will be entered into the project. Subgroup analysis will be performed for potential effect modifiers.

To assess whether protocol deviations or care provided outside the intervention have caused bias, the results of the intention-to-treat analysis will be compared to the on-treatment analysis.

#### Prognosis of therapy outcome

To identify the prognostic outcome variables on upper extremity functioning, a regression analysis will be performed on the group which received mental practice training. The WMFT scores at 6 and 12 months will be introduced as the dependent variable in the model, and the following hypothesized predictors as independent variables: age, sex, brain lesion site, ability to imagine motor acts (Vividness of Movement Imagery Questionnaire; VMIQ), cognitive level (Cog-log), credibility and expectancy of training (CEQ), general arm muscle strength (WMFTtask_strength _on T0) (as presented in Table 3). The model will be adjusted for possible baseline differences on the WMFT.

**Table 3:** Hypothesized predictors for treatment outcome

- age
- sex
- brain lesion site
- training expectancy and credibility
- ability to imagine motor acts
- cognitive level
- general arm muscle strength

#### Process evaluation

Entries in the patients' diaries and both the process evaluation of the patient and the occupational therapist will be analyzed descriptively.

#### Economic evaluation

Incremental ratios of differences in costs to outcomes between the intervention and control groups will be calculated. Outcomes for an incremental cost-effectiveness ratio will be changes in upper limb function primarily measured by the WMFT. QALYs generated through the utility values estimated by a specific QoL measure will be used for the incremental cost-utility ratio. These ratios will be plotted on the cost-effectiveness/utility plane to determine the economic use of one intervention over another. Level of confidence regarding economic determination will be quantified through non-parametric bootstrap simulations of the incremental ratios at the 2.5 and 97.5 percentile marks. The net-benefit framework will further validate this level by describing the acceptability probability of the net (monetary/health) benefit as a function of the ceiling ratio, or society's maximum willingness to pay. A 4-state Markov with 6-month cycle to approximate the time span between medical follow-ups will be used to simulate a long-term, 4-year period. Discounting of costs and outcomes will be undertaken at 4% [52]. Uncertainty about methodology and generalizability will be handled using a multi-way sensitivity analysis in which more than one study element (e.g., baseline subject characteristics and discounting) is varied.

## Discussion

In this study, the (cost) effectiveness of a mental practice-based training regime in improving arm function and daily activity performance in stroke patients will be evaluated. In addition, prognostic factors for a strong training outcome will be examined. A number of issues were taken into account in designing this study protocol.

Firstly, we intend to include tasks that would be appealing for all patients. For this reason, ordinary daily life activities have been chosen. Individual patients will be designated an activity that is currently just out of the reach of their functional abilities; this ought to trigger motivation. Reaching a point where they can perform the task will directly result in an improvement and facilitation of daily life, which will be the participants' reward for good practice. In order to maintain this cycle, the tasks' complexity level will constantly increase depending on improvement in upper extremity function.

Secondly, the current intervention training program was tested for feasibility. Patients who fulfilled the inclusion criteria and participated in an outpatient program of Heerlen's hospital department of rehabilitation medicine were included. After a variable baseline period with training as part of usual care, mental practice was added to the program. Patients trained at home were supervised by the rehabilitation team's occupational therapist. In a questionnaire, the compliance and feasibility of the training regime were scored. Participants appeared to experience no problems performing DVD-guided mental practice training guided twice a day for 7 days a week over 6 weeks, and reported good concentration levels during the 10-minute sessions. Only one participant, who showed rapid recovery in functional level, reported a decrease in motivational level. He was able to complete the most complex task (pouring water out of a can) in real life within the training period. Occupational therapists reported that the training program was feasible within the rehabilitation setting; these results were taken into account in designing the study protocol.

Thirdly, in choosing the current outcome measures, three different domains of the International Classification of Functioning (impairment, activity and participation) were included. The second domain – level of activity – was chosen as the primary outcome measure level, since changes in this domain seem most clinically relevant in daily life performance. Both the ability to perform daily activities (to be measured by the Wolf Motor Function Test) and the level of activity in a daily life situation (to be assessed using accelerometry), are included. In our opinion, additional assessment of real-world impaired arm activity by way of accelerometry is important, since discrepancies between motor capacity as measured by laboratory performance tests and its actual use in daily life can exist.

To conclude, this paper described the design of a randomized controlled clinical trial to study the effectiveness of mental practice training aimed at improving upper extremity functioning in stroke patients. The results of this study will provide evidence as to the (cost) effectiveness of the training as well as indicators for effective mental practice training in stroke patients.

## Competing interests

The author(s) declare that they have no competing interests.

## Authors' contributions

JAV is the main researcher and had an initiating role in writing this manuscript. HAS made important contributions during all phases of the study design. FRA designed the cost effectiveness part of the study. BHM, WWE and MMO had an important role in the design of the intervention and will have an important role in study-coordination on the various locations. All authors read and approved the final manuscript.

## Pre-publication history

The pre-publication history for this paper can be accessed here:


